# Role of M2 Muscarinic Receptor in the Airway Response to Methacholine of Mice Selected for Minimal or Maximal Acute Inflammatory Response

**DOI:** 10.1155/2013/805627

**Published:** 2013-04-18

**Authors:** Juciane Maria de Andrade Castro, Rodrigo R. Resende, Luciana Mirotti, Esther Florsheim, Layra Lucy Albuquerque, Adriana Lino-dos-Santos-Franco, Eliane Gomes, Wothan Tavares de Lima, Marcelo de Franco, Orlando Garcia Ribeiro, Momtchilo Russo

**Affiliations:** ^1^Department of Immunology, Institute of Biomedical Sciences, University of São Paulo, 05508-000 São Paulo, Brazil; ^2^Cell Signaling and Nanobiotechnology Laboratory, Department of Biochemistry and Immunology, Federal University of Minas Gerais, Minas Gerais, 31270-901 Belo Horizonte, Brazil; ^3^Laboratory of Imunogenetics, Instituto Butantan, 05503-900 São Paulo, Brazil; ^4^Department of Pharmacology, Institute of Biomedical Sciences, University of São Paulo, 05508-900 São Paulo, Brazil

## Abstract

Airway smooth muscle constriction induced by cholinergic agonists such as methacholine (MCh), which is typically increased in asthmatic patients, is regulated mainly by muscle muscarinic M3 receptors and negatively by vagal muscarinic M2 receptors. Here we evaluated basal (intrinsic) and allergen-induced (extrinsic) airway responses to MCh. We used two mouse lines selected to respond maximally (AIRmax) or minimally (AIRmin) to innate inflammatory stimuli. We found that in basal condition AIRmin mice responded more vigorously to MCh than AIRmax. Treatment with a specific M2 antagonist increased airway response of AIRmax but not of AIRmin mice. The expression of M2 receptors in the lung was significantly lower in AIRmin compared to AIRmax animals. AIRmax mice developed a more intense allergic inflammation than AIRmin, and both allergic mouse lines increased airway responses to MCh. However, gallamine treatment of allergic groups did not affect the responses to MCh. Our results confirm that low or dysfunctional M2 receptor activity is associated with increased airway responsiveness to MCh and that this trait was inherited during the selective breeding of AIRmin mice and was acquired by AIRmax mice during allergic lung inflammation.

## 1. Introduction

The cholinergic system plays a role in the regulation of many pathophysiological mechanisms. In the lung, the vagal parasympathetic nervous system via muscarinic receptors represents the dominant autonomic control of airway smooth muscle tone. Acetylcholine released at neuromuscular junctions binds to M3 muscarinic receptors in the smooth muscle and promotes airway contraction through a number of well-defined intracellular signaling mechanisms [1, 2]. An important negative feedback mechanism is given by the neuronal M2 muscarinic receptor that inhibits acetylcholine release [3, 4]. Airway hyperresponsiveness (AHR) is an exaggerated smooth muscle constriction observed in certain individuals among a population, which can occur in response to a variety of stimuli such as histamine, exercise, cold air, and methacholine (MCh). AHR is a cardinal feature of asthma [5]. Although there are exceptions [6], studies in asthmatic patients indicate a positive correlation between AHR and allergic eosinophilic inflammation [7]. Accordingly, susceptibility to develop asthma is associated with IL-5, a key cytokine for eosinophil differentiation, activation, and survival [8, 9]. One of the proposed mechanisms for allergic AHR to MCh is loss of function of vagal M2 receptors caused by eosinophilic inflammation [10–14]. Indeed, blockage of M2 receptors results in an increased acetylcholine release from vagal nerve endings that ultimately enhances bronchoconstriction [4, 15]. In this sense, AHR can be viewed as an integrated immune-neural circuit. Another well-established immune-mediated neural circuit is the cholinergic anti-inflammatory pathway [16–18]. This efferent pathway is triggered by proinflammatory cytokines via afferent sensory neurons and culminates in the release of acetylcholine by the vagus nerve. In turn, acetylcholine signals thorough nicotine acetylcholine receptor subunit *α*7 (*α*7nAChR), expressed on immune cells, inhibiting proinflammatory cytokine production [19]. 

Because the cholinergic system is involved in bronchoconstriction as well as regulation of inflammation, the present study aimed to evaluate the airway responses to MCh and airway allergic responses in two mouse lines selected to respond maximally (AIRmax) or minimally (AIRmin) to an innate inflammatory stimuli [20], focusing mainly on the role of M2 receptors. These mouse lines were produced by bidirectional selective breeding from a genetically heterogeneous population and behave differently in several experimental models such as arthritis [21], lung cancer [22], and bacterial infection [23]. Here we evaluated whether an innate response that selected the gene combinations determining AIRmax and AIRmin phenotypes would affect basal (intrinsic) or allergic (extrinsic) airway responses to MCh as well as an immune-adaptive T-helper-type-2- (Th2-) mediated stimuli in an experimental model of lung eosinophilic inflammation. 

## 2. Results 

### 2.1. Respiratory Pattern and Expression of M2 and M3 Muscarinic Receptors

**Figure 1:**
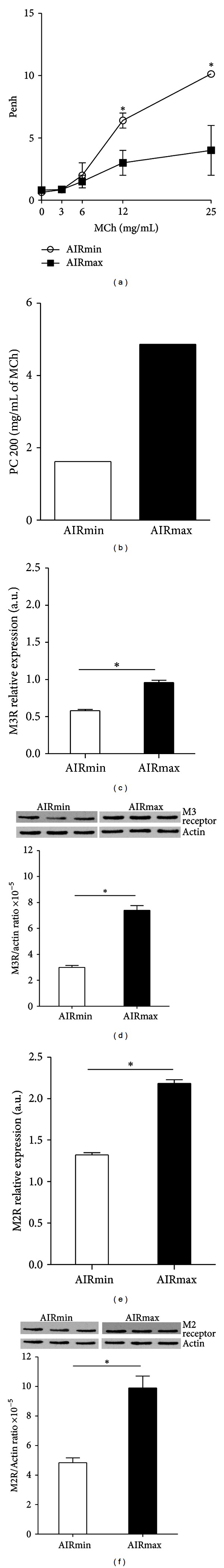
Respiratory pattern and expression of muscarinic receptors in AIRmin and AIRmax mice. Respiratory pattern was determined in awake, unrestrained mice by noninvasive whole-body barometric plethysmography. (a) Penh values were used as an index of bronchoconstriction induced after sequential delivery of increasing concentrations of MCh (3, 6, 12, and 25 mg/mL) and (b) provocative concentration of aerosol MCh at a 200% increase (PC200) over baseline values. Gene (c and e) or protein (d and f) expression of M2 and M3 muscarinic receptors was evaluated by real-time PCR or Western blot analysis in lungs from AIRmin and AIRmax mice. Real-time PCR was carried out using *β*-actin gene expression as internal control for normalization of M3R (c) and M2R (e) mRNA transcription levels. All PCR reactions were quantitative reactions made by real-time PCR. In Western blot analysis the density of M3R (d) and M2R (f) protein expression was nor aerosol at a malized to actin expression in each sample. Western blots were quantified by densitometry using the ImageJ software (NIH). Real-time PCR and Western-blot analyses were performed using pooled lungs from 5 mice. Data are expressed as mean ± SEM of five mice per group and are representative of three experiments; *P < 0.05.

It was previously reported that the basal airway responsiveness varies markedly among inbred strains of mice [24, 25]. Indeed, under basal noninflammatory condition multiple quantitative trait loci linked to AHR were identified in inbred mice strains [26, 27]. Therefore, we first determined the basal respiratory pattern of AIRmin and AIRmax in response to increasing doses of MCh in conscious unrestrained mice using a noninvasive whole-body barometric plethysmography. AIRmin mice showed a marked increase in Penh values when compared to AIRmax mice (Figure 1(a)). The dose of MCh required to elicit a 200% increase over baseline values (PC200) was 2.5-fold higher in AIRmax than in AIR min (Figure 1(b)). Our results indicate that the genetic selective process markedly influenced airway response to MCh. In this context, AIRmin animals can be considered as hyperresponsive whereas AIRmax animals are hyporresponsive to MCh. 

The contractile response of airway smooth muscles to muscarinic agonists is induced mainly by activation of muscle M3 receptor subtype and inhibited by activation of vagal M2 receptor subtype. Thus, we determined the expression of M3 and M2 muscarinic receptors in lung tissue. We found that M3 receptor expression was lower in AIRmin than in AIRmax animals, as revealed by real-time PCR and Western blot analysis (Figures 1(c)-1(d)), thus, it could not explain the hyperresponsiveness of AIRmin mice to MCh. Therefore, we evaluated M2 muscarinic receptor expression by PCR and Western blot analyses. M2 receptor expression was lower in AIRmin than in AIRmax mice (Figures 1(e)-1(f)). Altogether, these results indicate that low expression of muscarinic M2 receptor in AIRmin mice might be associated with the increased AHR to MCh observed in these animals.

### 2.2. *In Vivo* M2 Muscarinic Receptor Activity

**Figure 2:**
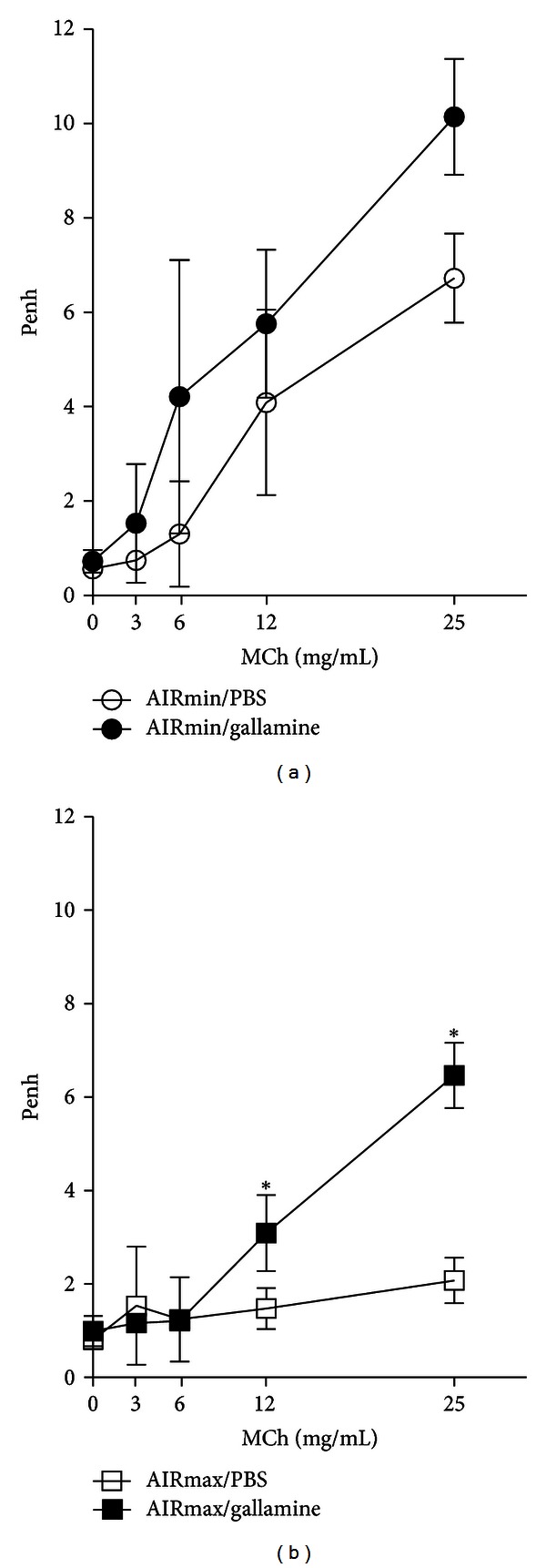
Respiratory pattern and airway resistance of AIRmin and AIRmax mice in the presence or absence of gallamine, a specific M2 receptor antagonist. Respiratory pattern to inhaled MCh was measured using noninvasive method in AIRmin (a) and AIRmax (b) mice. Airway resistance was measured by invasive method in AIRmin (c) and AIRmax (b) mice. Measurements were performed in the presence (black symbols) or absence (open symbols) of gallamine, in response to increasing concentrations of MCh. Results are reported as Penh values (a-b). Penh data represent the means ± SEM of five mice per group and are representative of three experiments (unpaired *t*-test, *P < 0,05 as compared to PBS group).

To evaluate the role of M2 receptor subtype on the airway responses *in vivo, *we treated the animals with gallamine, a selective M2 receptor antagonist. Gallamine treatment increased significantly Penh values in AIRmax but not in AIRmin mice when compared with untreated mice (Figures 2(a)-2(b)). However, gallamine treatment increased the responses to MCh in AIRmin animals indicating some activity of M2 receptor, but this activity was not sufficient to generate statistically significant data when compared to untreated group. In our study, we only used one dose of gallamine based on a previous report [28]. Taken together, these results indicate that the negative modulatory effect exerted by vagal M2 receptors was present in AIRmax mice but minimally in AIRmin animals. Therefore, the robust response to MCh observed in AIRmin mice and the lack of significant effect of gallamine indicate dysfunctional vagal M2 activity in this mouse line. 

### 2.3. *Ex Vivo* M2 Receptor Activity

**Figure 3:**
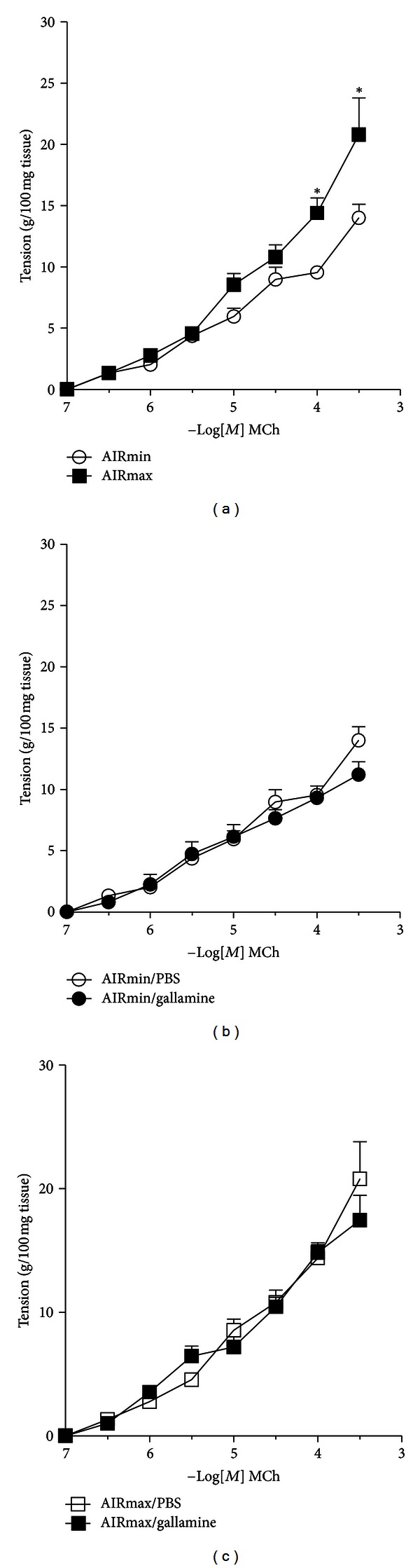
Tracheal responsiveness to MCh in AIRmin or AIRmax mice. Tracheal responsiveness of AIRmin or AIRmax to inhaled MCh was evaluated in the absence (a) or presence (b and c) of gallamine. Mouse tracheal segments were cultured in PBS or with gallamine for 20 min. Thereafter, the segments were maintained in the organ bath, and the contractile responses to MCh were recorded. Data are expressed as mean ± SEM of six mice per group *P < 0.05.

The results obtained *in vivo* with the noninvasive method indicated a major role of vagal M2 receptors in inhibiting airway constriction. In order to determine the role of muscle M2 receptors, we evaluated *ex vivo *the effect of gallamineonsmooth muscle constriction to MCh in isolated trachea. In this scenario, vagal receptors are excluded, and only muscarinic receptors of airway smooth muscle responded to MCh. We found that tracheal smooth muscle constriction induced by MCh was more intense in AIRmax when compared with AIRmin animals (Figure 3(a)). Gallamine treatment did not affect tracheal smooth muscle constriction induced by MCh (Figures 3(b)-3(c)), indicating that muscle M2 receptors have no role in smooth muscle contraction induced by MCh. 

### 2.4. Allergic Lung Inflammation and Expression of Muscarinic Receptors

**Figure 4:**
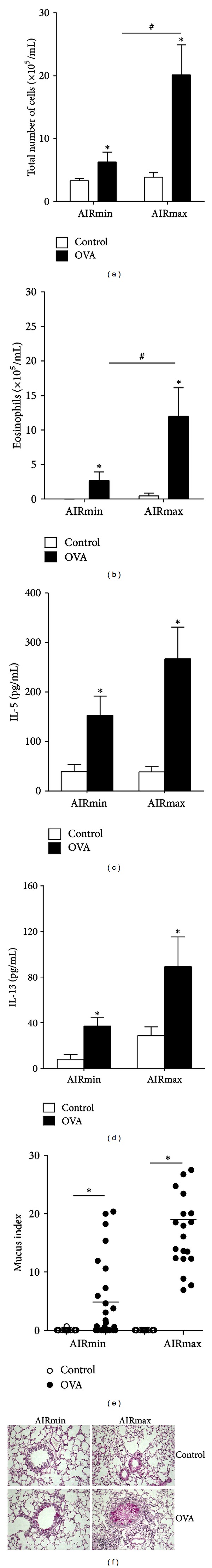
Allergic lung inflammation in AIRmin and AIRmax mice. AIRmin or AIRmax mice were sensitized twice (s.c.) with ovalbumin adsorbed on alum and challenged twice with ovalbumin (i.n.) (OVA group). Control group consisted of nonmanipulated animals. The experiments were performed 24 h after the last OVA challenge. The total inflammatory cells numbers (a), the number of eosinophils (b), and the levels of cytokines IL-5 (c) and IL-13 (d) were determined in bronchoalveolar lavage (BAL) fluid. The mucus index was determined by histocytometry of lung sections stained with periodic acid-Schiff (e) representative lung micrographs stained with HE of control and allergic AIRmin or AIRmax mice (original magnification ×200) (f). Data are expressed as mean ± SEM of four mice per group and are representative of two experiments; Statistical analyses of Student's *t*-test for (a), (b), (c), and (d). Statistical analyses of Mann Whitney-test for (e). *P < 0.05 relative to control group. ^#^ *P* < 0.05 relative to AIRmin mice.

Loss of M2 receptor function due to allergic inflammation has been well documented [29, 30]. It has been shown that eosinophilic major basic protein plays a key role in M2 receptor dysfunction (for review see [10]). We first determined the magnitude of allergic inflammation developed in AIRmin and AIRmax mice referred as OVA group.  Figure 4 shows that allergic lung inflammation, as revealed by total number of inflammatory cells (Figure 4(a)) and eosinophils (Figure 4(b)) in BAL, was more intense in AIRmax than in AIRmin animals. Accordingly, the levels of Th2 cytokines (IL-4 and IL-13) and mucus production were higher in AIRmax than in AIRmin mice (Figures 4(c)–4(e)). Histological analysis confirmed the more intense allergic inflammation in AIRmax when compared with AIRmin mice as shown by peribronchovascular cellular infiltrate and increased mucus (Figure 4(f)). 

**Figure 5:**
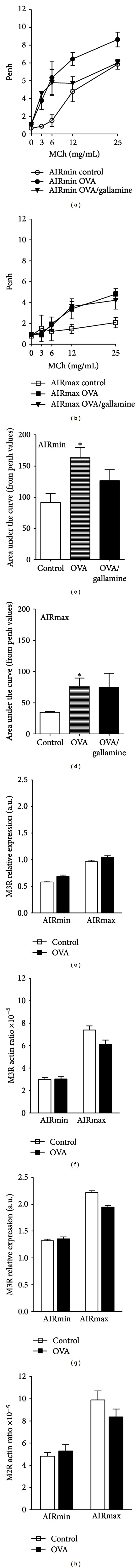
Pulmonary muscarinic receptors function and expression in allergic AIRmin and AIRmax mice. AIRmin or AIRmax mice were sensitized and challenged with OVA as in Figure 4. Control group consisted of nonmanipulated animals. The experiments were performed 24 h after the last OVA challenge. Respiratory pattern of allergic AIRmin (a and c) or AIRmax (b and d) to inhaled MCh was evaluated in the presence or absence of gallamine. Penh values were used as an index of bronchoconstriction induced after sequential delivery of increasing concentrations of MCh. Area under the curve was obtained from Penh values (c and d). Gene (e and g) or protein (f and h) expression of the M2 and M3 muscarinic receptors was evaluated by real-time PCR or Western blot analysis in lungs from OVA-sensitized AIRmin and AIRmax mice. The real-time PCR was carried out using *β*-actin gene expression as internal control for normalization of M3R (e) and M2R (g) mRNA transcription levels. In Western blot analysis the density of M3R (f) and M2R (h) protein expression was normalized to actin expression in each sample. Data are expressed as mean ± SEM of four mice per group and are representative of two experiments. Western blots data were quantified by densitometry using the ImageJ software (NIH). Statistical analyses of Student's *t* test for (a), (b), (e), (f), (g), and (h). Statistical analyses of ANOVA following Tukey HSD for (c) and (d). *P < 0.05 relative to control group; NS, not significant.

We next determined the respiratory pattern of allergic AIRmin and AIRmax animals in the absence or presence of gallamine. Firstly, we found that allergic AIRmin and AIRmax mice showed higher Penh values when compared with control mice (Figures 5(a)-5(b)). Statistical analysis performed on data, represented by area under the curve, confirmed that the responses to MCh were significantly increased in allergic mice when compared with control nonallergic mice (Figures 5(c) and 5(d)). However, no significant differences were observed in Penh values of allergic mice treated or not with gallamine (Figures 5(a)–5(d)). 

Because allergic mice showed an enhanced response to MCh when compared with control mice and gallamine treatment was ineffective in increasing Penh values, we asked whether the expression of muscarinic receptors has changed during allergic inflammation. We found that allergic inflammation did not change the expression of M2 or M3 receptors (Figures 5(e)–5(h)). Altogether, these data suggest that M2 muscarinic receptors dysfunction rather than altered muscarinic expression might be responsible for the increased response to MCh observed in allergic AIRmax or AIRmin mice. 

## 3. Discussion

**Figure 6:**
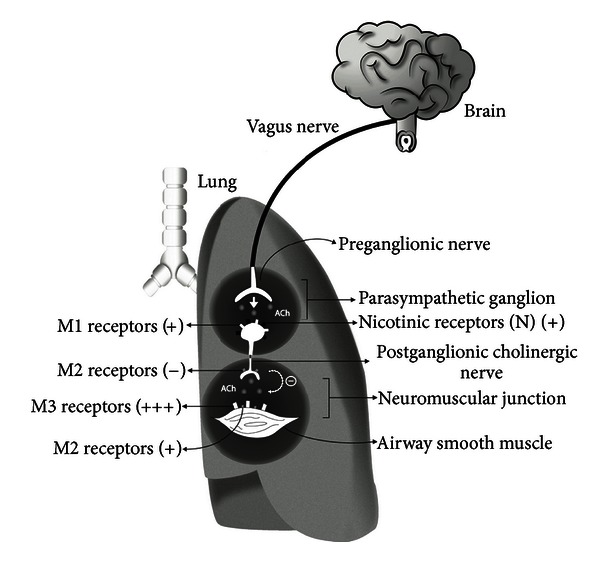
A model illustrating cholinergic receptors on pulmonary parasympathetic nerves and airway smooth muscle. Acetylcholine (ACh) released at parasympathetic ganglion binds to nicotinic (N1) and muscarinic (M1) receptors and activates postganglionic cholinergic nerve to release ACh at neuromuscular junction. ACh at neuromuscular junctions binds to M3 receptors in the airway smooth muscle, inducing muscle contraction, and to the autoinhibitory M2 receptors located in the vagus nerve, that limits ACh release at the neuromuscular junction and decreasing smooth muscle constriction.

Our work shows that AIRmin and AIRmax mouse lines represent a model to study lung muscarinic receptor functions and its relation with the development of an allergic lung disease. The main cholinergic pathways are depicted in Figure 6. We found that AIRmin and AIRmax animals develop different patterns of airway reactivity under basal or inflammatory conditions. Notably, respiratory responses to increasing doses of MCh observed in AIRmin mice in basal condition were significantly higher than that obtained in AIRmax animals. Previous studies identified multiple quantitative trait loci (QTL) linked to basal, noninflammatory airway hyperreactivity in specific inbred mouse strains [26, 27, 31]. It was found in A/J mice that the enhanced baseline airway reactivity was independent of lymphocytes or bone marrow-derived cells [32]. Airway responses to MCh are regulated by muscarinic M3 receptor present on muscle cells, which induces muscle constriction, and negatively regulated by the activation of muscarinic M2 receptors on vagal nerve endings, which reduce acetylcholine release at the neuromuscular junction [2, 3, 33, 34]. Here we present evidence that enhanced basal (intrinsic) airway reactivity of AIRmin mice is associated with the low expression and activity of M2 receptors. This assumption is based on the following findings: firstly, mRNA and protein expression of M2 receptors were decreased in AIRmin mice compared to AIRmax animals; secondly, although mRNA and protein expression of M3 receptors, the major muscarinic pathway for airway smooth muscle constriction, were increased in AIRmax mice, these animals did not show enhanced basal airway reactivity; thirdly, the use of gallamine, a selective M2 receptor antagonist significantly increased the airway responses of AIRmax mice, but affected minimally the responses of AIRmin mice. These results indicated that gallamine treatment impaired M2 function in AIRmax but not in AIRmin mice. Importantly, in conditions where the participation of vagal M2 receptors were precluded such as in *ex vivo* experiments with isolated tracheal rings, the tracheal responsiveness of AIRmin mice to MCh was not affected by gallamine treatment, reinforcing the predominant role of M3 receptors in smooth muscle constriction. Our *ex vivo* experiments with tracheal rings are in line with previous studies in M2 receptor KO mice, which showed that M2 receptors do not exert an inhibitory effect on tracheal constriction induced by cholinergic stimuli [35, 36]. Several studies have shown that the loss of M2 receptor function increases acetylcholine release and potentates vagally mediated bronchoconstriction (reviewed in [15]). Similar to our findings, experiments performed with estrogen receptor-*α* KO mice showed enhanced airway responsiveness to inhaled MCh and serotonin under basal conditions and this was associated with reduced M2 receptor expression and function [28, 36]. In addition, our results are in line with the work of Fisher et al. showing that M2-deficient (KO) mice display a significantly enhanced *in vivo* bronchoconstrictor response to vagal stimulation [37]. 

In allergic conditions, AIRmin and AIRmax mice showed a significant increase over baseline response (control group) in Penh value. The change in respiratory pattern of AIRmax animals after allergic inflammation could not be attributed to increased expression of M3 receptors. Because gallamine treatment had no effect, we favor the notion that the major mechanism for the increased respiratory responses of allergic AIRmax mice was due to decreased expression and/or dysfunction M2 receptor. This finding is in accordance with previous studies in allergic mice or asthmatic patients indicating that M2 receptor dysfunction rather than M3 receptor overexpression is associated with airway hyperreactivity (reviewed in [3, 4, 33]). It was shown by Ricci et al. (2002, 2008) that patients with asthma or rhinitis present different expression M2 and M3 receptors on lymphocytes, but the role of these receptors in these cells is still elusive [38, 39]. 

A striking difference between AIRmin and AIRmax animals was related to the intensity of T-helper-type-2-cells- (Th2-) mediated allergic responses such as eosinophilic inflammation, type 2 cytokines production, and mucus production that were all significantly higher in AIRmax when compared with AIRmin animals. It is known that inflammatory mediators play a role in enhancing bronchoconstriction by several pathways. The major basic protein from eosinophils can bind directly to M2 receptors, blocking the interaction with acetylcholine that leads to a higher release of acetylcholine in airway smooth muscles [10]. In addition, type 2 cytokines play a critical role in the development of bronchial responsiveness. IL-13, for instance, may increase bronchoconstriction by directly modulating smooth muscle contractibility and promoting airway hyperreactivity through multiple molecular mechanisms [40, 41]. The participation of these signaling pathways in our asthma murine model in AIRmin and AIRmax animals remains to be determined.

Interestingly, AIRmin and AIRmax mice that were selected by an innate stimuli maintained the polarized inflammatory profile with a stimulus derived from T-cell-dependent adaptive inflammation. We speculate that the enhanced Th2 responses of AIRmax animals was due to an enhanced production of inflammatory cells in bone marrow, as shown previously for neutrophils [42]. 

How M2 receptors could be associated with two contrasting situations? On one side, M2 dysfunction was associated with AIRmin animals that have been selected for low inflammatory response and on the other side with AIRmax animals that develop an intense allergic inflammation. These apparently conflicting findings can be reconciled by considering that acetylcholine can also regulate inflammation via the nicotinic acetylcholine receptor subunit alpha7 (*α*7nAchR) known as the “cholinergic anti-inflammatory pathway” [16, 17, 43, 44]. It follows that cholinergic anti-inflammatory pathway would favor the selective process of AIRmin animals. The *α*7nAchR is coded by *Chrna7* gene, which is mapped closed to inflammatory response modulator 1 (*Irm1*) locus on chromosome 7 [45]. The *Irm1* locus region contains about 230 known genes that are involved in inflammatory response regulation [45]. Interestingly, AIRmin mice present the same *Imr1* locus haplotype as A/J MCh hyperresponsive mice. The genotype of AIRmin at the rs32017050 (SNP at the peak of the *Irm1* locus) is “TT” similar to A/J mice, which is one of the 8 parental strains used in F0 population of those selected mice. On the other hand, AIRmax mice are “CC”, showing identical genotypes of C57BL/6J, CBA/J, DBA/2J, P/J, and SWR/J mice (mouse genomic informatics) that are hyporesponsive to MCh. Thus, M2 receptor dysfunction in AIRmin animals can be viewed as an additional mechanism inherited during the selective process that contributed for the low inflammatory phenotype. In the same vein, the loss of M2 receptor function observed in AIRmax animals during allergic inflammation could be interpreted as a neural homeostatic mechanism of the organism to control allergic inflammation [46]. Overall, the neuronal circuits that operate in inflammation or allergic asthma can be viewed as homeostatic mechanisms to counterbalance the inflammatory process. 

Since asthma is a complex syndrome, the AIRmin/AIRmax model might mirror clinical cases where airway reactivity is more prominent than airway inflammation as described in fatal asthma and in clinical conditions where airway inflammation is more robust than airway reactivity [47]. 

## 4. Material and Methods 

### 4.1. Mice

The AIRmax and AIRmin original mice were obtained by bidirectional selective breeding starting from a high polymorphic population produced by intercross between eight inbred mouse strains (A, DBA-2, P, SWR, CBA, SJL, BALB/c, and C57Bl/6) [20]. The selective breeding of these animals for maximal or minimal acute inflammatory response was based on both cellular infiltrate and protein contents in the inflammatory exudate 24 h after subcutaneous implant of polyacrylamide beads (Biogel). The main interline difference was characterized by the strong differential neutrophil PMN count in inflammatory exudate. Several quantitative trait loci (QTL) were recently detected in these mouse lines [46, 48]. Animals were obtained and maintained at the animal facilities of the Immunogenetics Laboratory at Butantan Institute on standard pellet food and water *ad libitum*. All experiments were performed following the guidelines for animal use approved by the Ethics Committee in Animal Experimentation of the University of São Paulo, Brazil, which is in accordance with the Ethical Principals of the Brazilian College of Animal Experimentation (COBEA), ethics protocol number 26, page 30, Book 2, ICB, USP. 

### 4.2. Ovalbumin (OVA) Sensitization and Challenge

Mice were sensitized and boosted subcutaneously (s.c.) with 4 *μ*g of chicken OVA (Sigma-Aldrich, St. Louis, MO, USA) and 1.6 mg of aluminum hydroxide gel in 0.2 mL of sterile PBS on days 0 and 7. Removal of lipopolysaccharides from OVA was performed as previously described [49]. After LPS removal, the endotoxin level of OVA was below the limit of detection in the LAL (Limulus amoebocyte lysate) assay (QCL-1000 kit, BioWhittaker, Walkersville, MD, USA). Airway inflammation was induced by two intranasal (i.n.) challenges with 10 *μ*g OVA on days 14 and 21, animals were anaesthetized with ketamine (50 mg/kg body weight) and xilazine (20 mg/kg body weight) intraperitoneally in 200 *μ*L of PBS for intra-nasal instilations. Experiments were performed 24 h after the last i.n. OVA challenge, on day 22.

### 4.3. Airway Responsiveness to Methacholine by Noninvasive Barometric Plethysmography

Respiratory parameters were determined before and after administration of increasing doses of inhaled MCh (3, 6, 12, and 25 mg/mL) by noninvasive method in conscious unrestrained mice using whole-body plethysmograph (Buxco Electronics Inc. Wilmington, North Carolina, USA), as previously described [50, 51]. Signals were analyzed using BioSystem XA software to derive whole-body flow parameters including respiratory frequency, tidal volume, minute ventilation, peak inspiratory flow, peak expiratory flow, and enhanced pause (Penh). Penh is a dimensionless value that takes into account box pressure recorded during inspiration and expiration and the timing comparison of early and late expiration, which was used to define the breathing pattern. Linear interpolation was used to determine the provocative concentration of MCh aerosol at which a 200% increase (PC200) over baseline values was observed for Penh values. To test M2 muscarinic receptor function, mice were exposed or not to M2 muscarinic receptor antagonist gallamine (30 *μ*M/animal) [28], nebulized 30 minutes before recording of respiratory parameters. 

### 4.4. *Ex Vivo* Trachea Responsiveness to Methacholine

To test M2 muscarinic receptor function, rings of tracheal tissue were removed and mounted using two steel hooks in a 15 mL organ bath for the measurement of isometric force contraction [47] in presence or absence of the M2 muscarinic receptor antagonist gallamine (30 *μ*M), added 20 minutes before administration of MCh. The force contraction was determined using a force displacement transducer and a chart recorder (Powerlab Labchart, AD Instruments, Colorado Springs, CO, USA). The tracheal tissue was maintained in an organ bath filled with Krebs-Hanseleit (KH) buffer at 37°C continuously aerated (95% O_2_ and 5% CO_2_) for 40 min (equilibrium period). After this period, the tracheal tension was adjusted to 0.5 g, and the tissue viability was assessed by replacing KH solution with KCl buffer (60 mM). The cumulative dose-response curve to MCh was constructed according to Van Rossum (1963) [52].

### 4.5. Bronchoalveolar Lavage Fluid

Mice were deeply anaesthetized by intraperitoneal injection of xilazine (50 mg/kg body weight) and ketamine (100 mg/kg body weight), folowed by cervical dislocation. Blood samples from the retro-orbital plexus were collected for serum antibody determinations. The trachea was cannulated, and lungs were washed twice with 0.5 and 1.0 mL PBS, respectively. Total and differential cell counts of bronchoalveolar lavage (BAL) fluid were determined by haemocytometer and cytospin preparation stained with Instant-Prov, hematoxylyn-eosin (Newprov, Pinhais, PR, Brazil).

### 4.6. Cytokines Measurements

The levels of cytokines (IL-5 and IL-13) in the BAL fluid were assayed by sandwich kit ELISA according to the manufacturer's instruction (PharMingen, San Diego, CA, USA), as previously described [53]. For IL-13 determinations, the pair used was 38 213.11 and biotinylated goat polyclonal anti-IL-13 from R&D Systems (Minneapolis, MN, USA). Values were expressed as pg/mL deduced from standards that run in parallel with recombinant cytokines. The limit of detection was 10 pg/mL for IL-5 and 31 pg/mL for IL-13.

### 4.7. Histological Analyses

After BAL collection, lungs were perfused via the right ventricle with 10 mL of PBS to remove residual blood, then immersed in 10% phosphate-buffered formalin for 24 h, and in 70% ethanol until embedded in paraffin. Lung sections with 5 *μ*m were stained with hematoxylin/eosin for evaluation of peribronchial and perivascular lung inflammation or with periodic acid-Schiff (PAS)/haematoxylin for the evaluation of mucus production, as previously described [51]. Briefly, a quantitative digital morphometric analysis was performed using the application program Metamorph 6.0 (Universal Images Corporation, Downingtown, PA, USA). The circumference area of bronchi and the PAS-stained area were electronically measured, and the mucus index was determined by the following formula: (PAS-stained area/bronchial circumference area) × 100.

### 4.8. Western-Blot Assays

Lungs from mice were homogenized in ice-cold lysis buffer containing protease inhibitors, 10 mM TrisHCl (pH 7.5), 8 M urea, 20 mM EDTA, 150 mM NaCl, and 1% Triton X-100. Membrane proteins (40 *μ*g) were fractionated by differential centrifugation, separated by SDS-PAGE and transferred to nitrocellulose membranes. The incubation procedure was performed using primary and secondary antibodies, as previously described [54–57]. The following polyclonal rabbit antisera were used: anti-M1 (1 : 200), anti-M2 (1 : 100), anti-M3 (1 : 100), and anti-*β*-actin (1 : 400) (Santa Cruz Biotechnology, Santa Cruz, CA, USA). *β*-actin protein expression was used as an internal standard for relative quantification of muscarinic receptors expression levels. Western blots were quantified by densitometry using the ImageJ software (NIH, Washington DC, USA).

### 4.9. RNA Isolation, Reverse Transcription, Real-Time PCR, and Conventional-PCR

Total RNA was isolated using Trizol (Invitrogen, Carlsbad, CA, USA)) from lungs of nonmanipulated AIRmax and AIRmin mice. Integrity of the isolated RNA was verified by separation of an aliquot of the extracted RNA on a 2% ethidium bromide-stained agarose gel. DNA was removed from RNA samples by incubation with DNase I (Ambion Inc., Austin, TX, USA). 

Total RNA (3 *μ*g) was reversely transcribed to cDNA with 200U of RevertAid H Minus M-MuLV-reverse transcriptase (Fermentas Inc., Hanover, MD, USA). DNA templates were amplified by real-time PCR on the 7000 Sequence Detection System (ABI Prism, Applied Biosystems, Foster City, CA,USA) using the Sybr green method [54, 56] or were amplified by PCR and analyzed as described previously [54, 56]. Variations in cDNA concentrations were normalized with *β*-actin as an internal control, which is a constitutively expressed gene. Experiments were performed in triplicate for each data point. Primer sequences for reverse transcription and quantitative PCR amplification (qRT-PCR) of mRNA used in this study were *β*-actin FWD ctg gcc tca ctg tcc acc tt, REV cgg act cat cgt act cct gct t; M2 mAChR FWD gct gcg tgg gtt ctt tcc t, REV ccc cta cga tga act gcc ag; M3 mAChR FWD cca tct ggc aag tgg tct tc, REV tgc cac aat gac aag gat gtt g. Negative controls were conducted on water and on total RNA. All PCR reactions were quantitative reactions, made by real-time PCR.

### 4.10. Statistical Analysis

Statistical analyses were performed employing JMP 9.0 (JMP SAS Institute Inc). Results are expressed as mean ± SEM. Continuous variables were log-transformed for the analyses when the normality of the distribution was rejected by the Shapiro-Wilk *W* test. For comparisons between two groups, normality was tested and then data were submitted to Student's *t*-test. For comparisons amongst 3 or more groups it was used ANOVA following In the case of absence of normality, even in log-transformed variables, Mann-Whitney was used for comparisons between 2 groups and or Kruskal-Wallis tests for three or more groups.

## References

[1] Fryer AD, Jacoby DB (1998). Muscarinic receptors and control of airway smooth muscle. *American Journal of Respiratory and Critical Care Medicine*.

[2] Roffel AF, Elzinga CR, Zaagsma J (1990). Muscarinic M3 receptors mediate contraction of human central and peripheral airway smooth muscle. *Pulmonary Pharmacology*.

[3] Fryer AD, Maclagan J (1984). Muscarinic inhibitory receptors in pulmonary parasympathetic nerves in the guinea-pig. *British Journal of Pharmacology*.

[4] Coulson FR, Fryer AD (2003). Muscarinic acetylcholine receptors and airway diseases. *Pharmacology and Therapeutics*.

[5] Boulet LP (2003). Physiopathology of airway hyperresponsiveness. *Current Allergy and Asthma Reports*.

[6] Crimi E, Spanevello A, Neri M, Ind PW, Rossi GA, Brusasco V (1998). Dissociation between airway inflammation and airway hyperresponsiveness in allergic asthma. *American Journal of Respiratory and Critical Care Medicine*.

[7] Brusasco V, Crimi E, Gianiorio P, Lantero S, Rossi GA (1990). Allergen-induced increase in airway responsiveness and inflammation in mild asthma. *Journal of Applied Physiology*.

[8] Bousquet J, Chanez DP,  Lacoste JY (1990). Eosinophilic inflammation in asthma. *New England Journal of Medicine*.

[9] Corren J (2011). Anti-interleukin-5 antibody therapy in asthma and allerges. *Current Opinion in Allergy and Clinical Immunology*.

[10] Jacoby DB, Gleich GJ, Fryer AD (1993). Human eosinophil major basic protein is an endogenous allosteric antagonist at the inhibitory muscarinic M2 receptor. *Journal of Clinical Investigation*.

[11] Hu J, Wang SZ, Forray C, El-Fakahany EE (1992). Complex allosteric modulation of cardiac muscarinic receptors by protamine: potential model for putative endogenous ligands. *Molecular Pharmacology*.

[12] Fryer AD, Jacoby DB (1992). Function of pulmonary M2 muscarinic receptors in antigen-challenged guinea pigs is restored by heparin and poly-L-glutamate. *Journal of Clinical Investigation*.

[13] Fryer A, Huang YC, Rao G (1997). Selective O-desulfation produces nonanticoagulant heparin that retains pharmacological activity in the lung. *Journal of Pharmacology and Experimental Therapeutics*.

[14] Elbon CL, Jacoby DB, Fryer AD (1995). Pretreatment with an antibody to interleukin-5 prevents loss of pulmonary M2 muscarinic receptor function in antigen-challenged guinea pigs. *American Journal of Respiratory Cell and Molecular Biology*.

[15] Belmonte KE (2005). Cholinergic pathways in the lungs and anticholinergic therapy for chronic obstructive pulmonary disease. *Proceedings of the American Thoracic Society*.

[16] Rosas-Ballina M, Tracey KJ (2009). Cholinergic control of inflammation. *Journal of Internal Medicine*.

[17] Tracey KJ (2002). The inflammatory reflex. *Nature*.

[18] Blalock JE (2002). Harnessing a neural-immune circuit to control inflammation and shock. *Journal of Experimental Medicine*.

[19] Blanchet MR, Israël-Assayag E, Cormier Y (2005). Modulation of airway inflammation and resistance in mice by a nicotinic receptor agonist. *European Respiratory Journal*.

[20] Ibanez OM, Stiffel C, Ribeiro OG (1992). Genetics of nonspecific immunity: I. Bidirectional selective breeding of lines of mice endowed with maximal or minimal inflammatory responsiveness. *European Journal of Immunology*.

[21] Peters LC, Jensen JR, Borrego A (2007). Slc11a1 (formerly NRAMP1) gene modulates both acute inflammatory reactions and pristane-induced arthritis in mice. *Genes and Immunity*.

[22] Maria DA, Manenti G, Galbiati F (2003). Pulmonary adenoma susceptibility 1 (Pas1) locus affects inflammatory response. *Oncogene*.

[23] Borrego A, Peters LC, Jensen JR (2006). Genetic determinants of acute inflammation regulate Salmonella infection and modulate Slc11a1 gene (formerly Nramp1) effects in selected mouse lines. *Microbes and Infection*.

[24] Levitt RC, Mitzner W (1988). Expression of airway hyperreactivity to acetylcholine as a simple autosomal recessive trait in mice. *The FASEB Journal*.

[25] Levitt RC, Mitzner W (1989). Autosomal recessive inheritance of airway hyperreactivity to 5-hydroxytyptamine. *Journal of Applied Physiology*.

[26] De Sanctis GT, Merchant M, Beier DR (1995). Quantitative locus analysis of airway hyperresponsiveness in A/J and C57BL/6J mice. *Nature Genetics*.

[27] De Sanctis GT, Daheshia M, Daser A (2001). Molecular mechanisms in allergy and clinical immunology: genetics of airway hyperresponsiveness. *Journal of Allergy and Clinical Immunology*.

[28] Carey MA, Card JW, Bradbury JA (2007). Spontaneous airway hyperresponsiveness in estrogen receptor-alpha-deficient mice. *American Journal of Respiratory and Critical Care Medicine*.

[29] Larsen GL, Fame TM, Renz H (1994). Increased acetylcholine release in tracheas from allergen-exposed IgE-immune mice. *American Journal of Physiology*.

[30] Fryer AD, Wills-Karp M (1991). Dysfunction of M2-muscarinic receptors in pulmonary parasympathetic nerves after antigen challenge. *Journal of Applied Physiology*.

[31] Ewart SL, Kuperman D, Schadt E (2000). Quantitative trait loci controlling allergen-induced airway hyperresponsiveness in inbred mice. *American Journal of Respiratory Cell and Molecular Biology*.

[32] Hadeiba H, Corry DB, Locksley RM (2000). Baseline airway hyperreactivity in A/J mice is not mediated by cells of the adaptive immune system. *Journal of Immunology*.

[33] Eglen RM, Hegde SS, Watson N (1996). Muscarinic receptor subtypes and smooth muscle function. *Pharmacological Reviews*.

[34] Fryer AD, Adamko DJ, Yost BL, Jacoby DB (1999). Effects of inflammatory cells on neuronal M2 muscarinic receptor function in the lung. *Life Sciences*.

[35] Stengel PW, Gomeza J, Wess J, Cohen ML (2000). M2 and M4 receptor knockout mice: muscarinic receptor function in cardiac and smooth muscle in vitro. *Journal of Pharmacology and Experimental Therapeutics*.

[36] Garssen J, Van Loveren H, Gierveld CM, Van der Vliet H, Nijkamp FP (1993). Functional characterization of muscarinic receptors in murine airways. *British Journal of Pharmacology*.

[37] Fisher JT, Vincent SG, Gomeza J, Yamada M, Wess J (2004). Loss of vagally mediated bradycardia and bronchoconstriction in mice lacking M2 or M3 muscarinic acetylcholine receptors. *The FASEB Journal*.

[38] Ricci A, Amenta F, Bronzetti E, Mannino F, Mariotta S, Khosrow Tayebati S (2002). Expression of peripheral blood lymphocyte muscarinic cholinergic receptor subtypes in airway hyperresponsiveness. *Journal of Neuroimmunology*.

[39] Ricci A, Mariotta S, Amenta F, Tayebati SK, Terzano C (2008). Changes in muscarinic cholinergic receptor expression in human peripheral blood lymphocytes in allergic rhinitis patients. *Pulmonary Pharmacology and Therapeutics*.

[40] Perkins C, Yanase N, Smulian G (2011). Selective stimulation of IL-4 receptor on smooth muscle induces airway hyperresponsiveness in mice. *Journal of Experimental Medicine*.

[41] Tliba O, Deshpande D, Chen H (2003). IL-13 enhances agonist-evoked calcium signals and contractile responses in airway smooth muscle. *British Journal of Pharmacology*.

[42] Ribeiro OG, Maria DA, Adriouch S (2003). Convergent alteration of granulopoiesis, chemotactic activity, and neutrophil apoptosis during mouse selection for high acute inflammatory response. *Journal of Leukocyte Biology*.

[43] Borovikova LV, Ivanova S, Zhang M (2000). Vagus nerve stimulation attenuates the systemic inflammatory response to endotoxin. *Nature*.

[44] Tracey KJ (2009). Reflex control of immunity. *Nature Reviews Immunology*.

[45] Vorraro F, Galvan A, Cabrera WHK (2010). Genetic control of IL-1*β* production and inflammatory response by the mouse Irm1 locus. *Journal of Immunology*.

[46] Gosens R, Zaagsma J, Meurs H, Halayko AJ (2006). Muscarinic receptor signaling in the pathophysiology of asthma and COPD. *Respiratory Research*.

[47] Wenzel SE (2012). Asthma phenotypes: the evolution from clinical to molecular approaches. *Nature Medicine*.

[48] Galvan A, Vorraro F, Cabrera W (2011). Association study by genetic clustering detects multiple inflammatory response loci in non-inbred mice. *Genes and Immunity*.

[49] Bortolatto J, Borducchi E, Rodriguez D (2008). Toll-like receptor 4 agonists adsorbed to aluminium hydroxide adjuvant attenuate ovalbumin-specific allergic airway disease: role of MyD88 adaptor molecule and interleukin-12/interferon-gamma axis. *Clinical and Experimental Allergy*.

[50] Rodríguez D, Keller AC, Faquim-Mauro EL (2003). Bacterial lipopolysaccharide signaling through Toll-like receptor 4 suppresses asthma-like responses via nitric oxide synthase 2 activity. *Journal of Immunology*.

[51] Keller AC, Mucida D, Gomes E (2006). Hierarchical suppression of asthma-like responses by mucosal tolerance. *Journal of Allergy and Clinical Immunology*.

[52] Van Rossum JM (1963). Cumulative dose-response curves. II. Technique for the making of dose-response curves in isolated organs and the evaluation of drug parameters. *Archives internationales de pharmacodynamie et de thérapie*.

[53] Russo M,  Nahori MA, Lefort J (2001). Suppression of asthma-like responses in different mouse strains by oral tolerance. *American Journal of Respiratory Cell and Molecular Biology*.

[54] Resende RR, Britto LRG, Ulrich H (2008). Pharmacological properties of purinergic receptors and their effects on proliferation and induction of neuronal differentiation of P19 embryonal carcinoma cells. *International Journal of Developmental Neuroscience*.

[55] Resende RR, Alves AS, Britto LRG, Ulrich H (2008). Role of acetylcholine receptors in proliferation and differentiation of P19 embryonal carcinoma cells. *Experimental Cell Research*.

[56] Resende RR, Gomes KN, Adhikari A, Britto LRG, Ulrich H (2008). Mechanism of acetylcholine-induced calcium signaling during neuronal differentiation of P19 embryonal carcinoma cells in vitro. *Cell Calcium*.

[57] Resende RR, Adhikari A, da Costa JL (2010). Influence of spontaneous calcium events on cell-cycle progression in embryonal carcinoma and adult stem cells. *Biochimica et Biophysica Acta*.

